# Gender differentials in the evolution of cigarette smoking habits in a general European adult population from 1993–2003

**DOI:** 10.1186/1471-2458-6-130

**Published:** 2006-05-12

**Authors:** Michael C Costanza, Julien Salamun, Alan D Lopez, Alfredo Morabia

**Affiliations:** 1 Division of Clinical Epidemiology, Geneva University Hospitals, 25, Rue Micheli-du-Crest, CH-1211 Geneva 14, Switzerland; 2 School of Population Health, University of Queensland, Herston Rd, Herston, 4006, Brisbane, Australia; 3 Center for the Biology of Natural Systems, Queens College-CUNY, 163-03 Horace Harding Expressway, Flushing, NY 11365, USA

## Abstract

**Background:**

Describe the recent evolution of cigarette smoking habits by gender in Geneva, where incidence rates of lung cancer have been declining in men but increasing in women.

**Methods:**

Continuous cross-sectional surveillance of the general adult (35–74 yrs) population of Geneva, Switzerland for 11 years (1993–2003) using a locally-validated smoking questionnaire, yielding a representative random sample of 12,271 individuals (6,164 men, 6,107 women).

**Results:**

In both genders, prevalence of current cigarette smoking was stable over the 11-year period, at about one third of men and one quarter of women, even though smoking began at an earlier age in more recent years. Older men were more likely to be former smokers than older women. Younger men, but not women, tended to quit smoking at an earlier age.

**Conclusion:**

This continuous (1993–2003) risk factor surveillance system, unique in Europe, shows stable prevalence of smoking in both genders. However, sharp contrasts in age-specific prevalence of never and former smoking and of ages at smoking initiation indicate that smoking continues a long-term decline in men but has still not reached its peak in women.

## Background

A literature review on changes in cigarette smoking by gender in different populations of the developed world after 1980 reveals that smoking is almost uniformly declining in men, but trends are much more diverse in women (Table 1) [1-15]. In most northern European and North American populations women are smoking less, but in most middle and southern European populations, including Switzerland, the prevalence of current smoking among women is still on the rise.

All the trends in Table 1 were based on a maximum of three assessments of population smoking habits during a decade. One aim of the present study was to capitalize on 11 years of continuous monitoring of the cigarette smoking habits of the population of Geneva, Switzerland to provide more reliable and stable trend estimates. A second aim was to determine whether the recent evolution of cigarette smoking habits by gender corresponds to specific stages of the smoking epidemic model proposed by Lopez et al [16], as well as to provide more recent empirical evidence to suggest how that model may need to be updated.

## Methods

### 11-year surveillance and study subjects

The *Bus Santé *("Health Bus") survey is an ongoing, community-based surveillance project designed to monitor chronic disease risk factors in Geneva, Switzerland continuously since 1993 [17]. Geneva (city and canton) has a population of about 420,000 primarily French-speaking inhabitants distributed over 242 km^2^. Survey subjects were selected independently each year since 1993 to constitute a random sample from the study populations of approximately 100,000 each of non-institutionalized men and women aged 35 to 74 years at the time of their interview.

Eligible subjects are identified from an annual residential database established by the local cantonal government using a standardized procedure. All legal residents are registered. The list includes all potential eligible participants except for persons living under illegal conditions. The only specific information from the list used in the survey (gender, age, and whether the person is of Swiss origin) is highly accurate. Stratified random sampling by gender within 10-year age strata is proportional to the corresponding population distributions. Selected subjects are mailed an invitation to participate and, if they do not respond, up to seven telephone calls at different times on various days of the week are made. If telephone contact is unsuccessful, two more letters are mailed. Each subject's recruitment lasts from two weeks to two months. Subjects not reached (15% of men, 19% of women) are replaced using the same selection protocol. Routine monitoring of survey quality had shown that these subjects no longer resided in the canton, so were not eligible for the study. Subjects who refuse to participate are not replaced. Participating subjects are not eligible in future surveys. Annual participation rates have ranged from 57 to 65%. Demographic characteristics and smoking status are obtained from non-participants to document selection bias.

Each participant receives several self-administered, standardized questionnaires covering exposure to risk factors for the major lifestyle chronic diseases, including cigarette smoking. During a scheduled appointment at a mobile epidemiology clinic (housed in a special bus) the questionnaires are checked for completion by trained health technicians. Self-reported current smoking had sensitivity 88.6% and specificity 87.2% when validated against concentrations of both salivary thiocyanate and expired carbon monoxide [18].

### Cigarette smoking outcomes

Survey participants were post-classified into cigarette smoking outcome subgroups based on their questionnaire responses. Never smokers reported having smoked less than 100 cigarettes in their lifetime. Current smokers reported having smoked during the year before their interview. Former smokers reported having quit smoking at least one year before their interview. Current and former smokers reported their age at smoking initiation, and the total duration of smoking was calculated. The intensity of cigarette smoking was summarized using the number of cigarettes smoked per day (CPD). The duration and intensity of cigarette smoking also were summarized jointly using pack-years (total duration × mean packs of 20 cigarettes/d). For both CPD and pack-years, log-transformed data were analyzed and the results were expressed as the geometric mean weighted over all smoking episodes.

### Statistical analyses

#### Trends by calendar year

Preliminary investigations of trend were based on multiple linear regression models in which each of the current, former, and never smoking subgroup individual-level (indicator) variables were regressed separately on the three main effects of gender (indicator variable), age (years), and year of survey in the presence of all possible subsets of corresponding 2- and 3-way interaction terms. The aim of these analyses was to determine whether simpler linear regression models stratified by gender would suffice to capture the main trend features of the data. Next, gender-specific models with and without adjustment for age were compared to determine if age-adjustment was required. The various regression coefficient *p*-values in all these analyses were employed as rough guidelines for identifying statistically significant main and interaction effects.

The final trend analyses were performed separately by gender without age-adjustment. Simple linear regression models were used to estimate annual cigarette smoking subgroup prevalences and (arithmetic or geometric) means (Y) based on the individual participant (un-aggregated) data, and the survey year (X) regression slope was used to measure annual change [19]. Corresponding simultaneous 95% confidence bands (CB) for the regression lines were obtained, respectively, by subtracting and adding the following quadratic expressions, to the linear regression estimated prevalences/means:

Q_Lower _= [ 2F_0.05/k_(2, n-2) * MSE * {1/n + (Y-mean Y)^2^/SS_X_} ]^1/2^,

Q_Upper _= [ 2F_1-(0.05/k)_(2, n-2) * MSE * {1/n + (Y-mean Y)^2^/SS_X_} ]^1/2^,

where n = sample size; k = the number of regression models for which CB were obtained; F_0.05/k_(2, n-2), F_1-(0.05/k)_(2, n-2) = (0.05/k)^th ^and (1-(0.05/k))^th ^percentiles of the central F distribution with 2 and (n-2) degrees of freedom; MSE = mean squared error from the linear regression; SS_X _= sum of squares of X about the mean X [20]. The linear regression-estimated annual measures were depicted graphically together with the corresponding annual raw measures to provide some idea of background sampling fluctuations. The corresponding *p*-value was used to test the null hypothesis that the regression slope = 0 (no trend). A *p*-value < 0.05 (< 0.10) was considered statistically (or borderline) significant for all trend tests conducted.

#### Age effects

Gender-specific age effects over all survey years combined for the three cigarette smoking subgroup prevalences were investigated with stratified histograms, and the mean ages at smoking initiation were investigated using simple linear regression models and graphs with 95% CB similar to those described above for the trends by calendar year analyses. The age subgroups [35–39], [40–44], [45–49], [50–54], [55–59], [60–64], [65–69], [70–74] years were employed in these analyses. Checks on possible period effects were made by re-examining the age effect results after stratification by the year of survey subgroups [1993–1994], [1995–1996], [1997–1998], [1999–2000], [2001–2003].

### Ethical approval

The study was approved by the Geneva University Ethics Committee. All study participants provided informed written consent.

## Results

In the preliminary trend analyses the prevalences of both former and never cigarette smoking interacted with all of gender, age, and year of survey (both 3-way *p *< 0.04, all 2-way *p *< 0.07), and there were main effects of gender (both *p *< 0.07), age (both *p *< 0.006), and year of survey (both *p *< 0.02). For current smoking prevalence there were no such main effects or interactions (all *p *> 0.75).

Moreover, in the ensuing analyses that were performed after stratification by gender, there was little difference between the crude and the age-adjusted year of survey trend effects (all *p *> 0.38) (data not shown otherwise). By design, the gender-specific age distributions of the sample participants were virtually identical across survey years. Thus, the final trend results described below were based on gender-specific simple linear regression models without age-adjustment.

Similarly, minor differences in the age effect analyses stratified by the period (year of survey) subgroups were entirely as expected due to annual survey sampling background variation (data not shown otherwise.) Hence, the final age effect results described below were collapsed over all survey years combined.

### Never cigarette smoking

**Figure 1:**
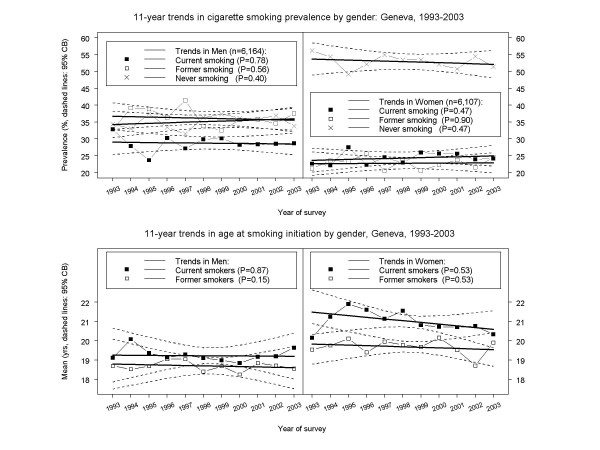
11-year trends in cigarette smoking habits by gender in Geneva, Switzerland, 1993–2003. All estimates based on analyses of individual-level data (6,164 men, 6,107 women). Symbols connected by piece-wise line segments: least squares means; bold solid lines: linear regressions; dashed curves: confidence bands (CB). Top panels: prevalence of current, former, and never cigarette smoking (95% CB's adjusted for six models). Bottom panels: age at smoking initiation for current and former cigarette smokers (95% CB's adjusted for four models).

**Figure 2:**
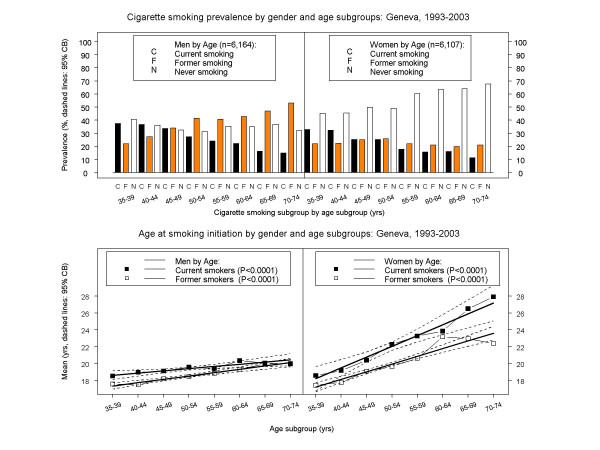
Cigarette smoking habits across five-year age subgroups by gender in Geneva, Switzerland, 1993–2003. All estimates based on analyses of individual-level data (6,164 men, 6,107 women). Bar heights: prevalences; symbols connected by piece-wise line segments: least squares means; bold solid lines: linear regressions; dashed curves: confidence bands (CB). Top panels: prevalence of current, former, and never cigarette smoking. Bottom panels: age at smoking initiation for current and former cigarette smokers (95% CB's adjusted for four models).

The prevalence of never smoking remained virtually unchanged from 1993 through 2003 in both genders (Figure 1, top panels). Over one third of men and over one half of women were never cigarette smokers. Never smoking declined through ages 50–54 years in men, but younger women were less likely to be never smokers than older ones (Figure 2, top panels).

### Current cigarette smoking

The prevalence of current cigarette smoking remained virtually unchanged from 1993 through 2003 in both genders (Figure 1, top panels). Around 28% of men and one quarter of women were current cigarette smokers. There was an almost identical decline with age in both genders (Figure 2, top panels).

There were no trends for current smokers of either gender in mean age at smoking initiation (Figure 1, bottom panels). Men began smoking at around age 19, and women at around age 21. However, younger men tended to start smoking earlier than older men (e.g., 18 vs. 20 yrs), while younger women tended to start smoking much earlier than older women (e.g., 18 vs. 28 yrs) (*p *< 0.0001, Figure 2, bottom panels).

There were no trends in cigarettes smoked per day (CPD) among current smokers of either gender. On average, men had smoked around 15–16 CPD, and women around 11–12 CPD. There were no associations of CPD with age in current cigarette smokers of either gender. Likewise, there were no trends for current smokers of either gender in pack-years smoked. On average, men had smoked around 20 pack-years, and women around 15 pack-years. (Data not shown otherwise.)

### Former cigarette smoking

The prevalence of former smoking remained virtually unchanged from 1993 through 2003 in both genders (Figure 1, top panels). Over one-third of men and almost one-quarter of women had quit smoking. However, there were very different age effects by gender. Among men there was a striking increase in former smoking by age, while among women former smoking remained relatively constant across all ages (Figure 2, top panels).

Former smoking men began smoking at around age 18.5 (similar to current smokers), while former smoking women began smoking at around age 19.5 (vs. age 21 for current smokers) (Figure 1, bottom panels). There were no trends by gender in age at smoking initiation among former smokers, but younger former smoking men began smoking earlier than older men, and younger former smoking women began smoking much earlier than older women (Figure 2, bottom panels).

Former smoking men had a borderline decreasing trend in CPD (from around 18 to 14 CPD, *p *= 0.054), but they still had smoked more than women former smokers, who had smoked around 10 CPD with little change. There were decreases in CPD with age for former smokers, borderline in men (*p *= 0.091) and significant in women (*p *= 0.0012). Former smoking men also showed a decreasing trend in the total amount smoked when they quit (from 12 to 10 pack-years, *p *= 0.0091), but still had smoked twice as much as women former smokers, who tended to quit after 6 pack-years. There was a 2-year decrease in smoking duration among men former smokers (from 20 to 18 yrs, *p *= 0.0064), but no trend among women former smokers, who had smoked for around 15 yrs. In both genders, smoking durations were 10–12 yrs less on average for former vs. current smokers. There was a decreasing trend in age at smoking cessation among former smoking men (from 39 to 36 yrs, *p *= 0.0032), but no trend among former smoking women. On average, men former smokers had quit smoking by age 38 yrs, and women former smokers had quit by age 34 yrs. (Data not shown otherwise.)

## Discussion

The present data show that current cigarette smoking remained stable in Geneva men and women from 1993 through 2003. This may superficially suggest that cigarette smoking patterns by gender are the same. However, there appear to be fundamentally different smoking behavior processes for men vs. women.

Among men, the smoking epidemic has declined persistently for at least 20 years, as evidenced by the high prevalence of former smokers among older men. Men now 70 to 75 years of age are mostly former smokers. Also, younger men tended to stop smoking after having smoked fewer pack-years than older men. Finally, men stopped smoking at an earlier age in 2003 compared to 1993. Whether this decline will continue is unknown, as current cigarette smoking remains more prevalent among younger men, who also began smoking at an earlier age than older men.

In sharp contrast, the smoking epidemic still seems to be on the rise among women. Compared to older women, younger women began smoking cigarettes at a much earlier age and smoked more. In addition, and despite the fact that current cigarette smoking prevalence was very similar across five-year age groups in both genders, the prevalence of former smoking was much lower among older women than older men. Moreover, absence of a decline in the age at smoking cessation among women indicates little will to stop smoking at the population level. Indeed, there was an increasing trend in pack-years smoked among women who had stopped smoking between 1993 and 2003.

The observed smoking decrease among men and the smoking increase among women are in general accord with lung cancer surveillance data collected by the Geneva Cancer Registry. Specifically, lung cancer incidence (per 100,000/yr, age-standardized to the EU population) for the years 1983–1986, 1995–1998, and 1999–2002, respectively, declined from 80.7 to 75.2 to 67.1 in men, and increased from 14.8 to 24.3 to 27.8 in women [[22] and personal communication].

In 1994, Lopez et al [16] proposed a model describing the dynamic of the cigarette smoking epidemic in developed countries that comprised four different developmental stages. In Stage I, smoking prevalence is low in both men and women, but begins to increase rapidly among men. In Stage II, smoking prevalence among men continues its rapid rise, reaching a peak between 50% and 80%, and the prevalence of former smoking is low in both genders; smoking prevalence among women lags behind that of men, but eventually begins to increase as rapidly as that for men in Stage I. In Stage III, smoking prevalence among men begins to fall and the proportion of former smokers, especially in the older age groups, starts to rise; smoking prevalence among women reaches a plateau somewhat below the peak of men in Stage II; and national/regional anti-smoking activities are adopted which attempt to modify perceptions of cigarette smoking from being a socially acceptable to an unacceptable behavior. In Stage IV, smoking prevalence for both genders falls to around 30% and continues to decline, but only slowly. Because of the latency period between exposure to tobacco smoke and death from tobacco-related causes, the evolution of smoking-related deaths, including lung cancer, follows a somewhat paradoxical path across the four stages. In men it reaches a peak in Stage III, but in women it continues to increase throughout Stage IV.

Different countries vary in terms of where they are with respect to the above four developmental stages. Because lung cancer incidence in the 1990's was steadily declining in men but increasing in women, the trends observed in Geneva over the past decade correspond quite closely to those characterized by Stage IV in the smoking epidemic model of Lopez et al [16]. However, there are also some important amendments to the model suggested by these more recent data. Firstly, smoking prevalence among women at the end of Stage IV is likely to be 4–5 percentage points lower than that for men, as opposed to 1–2% lower predicted by the model. Moreover, at the end of Stage IV, and presumably a dominant characteristic of an additional Stage V, smoking prevalence in both genders would change only marginally from year to year, less steeply than predicted by the smoking epidemic model.

### Study limitations and strengths

Recall bias in retrospectively reported age at smoking initiation is a potential study limitation. However, it has been found that adult self-reported age at first substance use, as routinely collected via survey questionnaires, is sufficiently reliable for most epidemiological applications [22,23]. In addition, the survey focus on adults 35–74 years of age is another potential study limitation since other interesting dynamics in the evolution of the tobacco epidemic may be occurring among younger women and men.

The ability to obtain 11-year trends based on annual independent surveys using the same sampling and data collection methodology is a study strength. The annual participation rates of 57 to 65%, while not ideal, are typical for this type of survey. Survey protocols and participation rates have been very stable across the years. The questions on cigarette smoking habits were included in a larger standardized questionnaire requesting information on a variety of risk factors in addition to smoking, and the study participants were post-classified into the three smoker subgroups solely on the basis of their questionnaire responses. For example, individuals who reported having quit smoking less than one year before their interview were classified as current smokers in the analyses. It is unlikely that any potential inaccuracies in reporting smoking behaviors would have affected the annual surveys differentially. Moreover, the distributions of never, former, and current cigarette smokers among survey participants vs. non-participants were not markedly different (data not shown otherwise). Likewise, major selection bias is unlikely as the study participants comprise a large, representative, random sample of the Geneva general adult population.

## Conclusion

The Geneva data suggest a marked slowdown in the rate of decline of smoking prevalence, typical of many other developed countries (see Table 1). Thus, substantial new efforts will be required to produce further reductions in smoking prevalence.

The smoking epidemic model of Lopez et al [16] may need to be updated if it is to be useful for guiding and reinforcing the need for further tobacco control efforts in the 21^st ^century. More studies are needed to determine whether the Geneva data are typical of current changes in the prevalence and mortality from cigarette smoking throughout the developed world.

## Competing interests

The author(s) declare that they have no competing interests.

## Authors' contributions

MCC designed, performed, and interpreted the analyses. JS performed and synthesized the literature search. ADL interpreted the results in the context of the smoking epidemic model. AM conceived and was responsible for the *Bus Santé *survey. All four authors contributed to drafting the manuscript, and read and approved the final manuscript.

## Pre-publication history

The pre-publication history for this paper can be accessed here:



## Figures and Tables

**Table 1:** Recent trends in current smoking by gender in various European populations. Countries are listed alphabetically within subgroups sorted by trend direction in women (increasing (↑), stable (↔), decreasing (↓)).

**Survey characteristics**		**Men**		**Women**		
*Country (type/place)*	*Year(s)*	*Trend direction*	*Smoking prevalence*	*Trend direction*	*Smoking prevalence*	[Reference]
Austria (National)	1986 1995	↑	35% 39%	↑	18% 24%	[1]
Belgium (Charleroi)	1985/87 1990/93	slight ↓	51% 48%	↑	24% 29%	[2]
Finland (Kuopio)	1982 1992	↓	34% 30%	↑	10% 13%	[2]
Finland (North Karelia)	1982 1992	↓	30% 27%	↑	9% 12%	[2]
France (National)	1974 1992	↓	59% 48%	↑	28% 33%	[3]
Germany (MONICA-Augsburg)	1985 1995	↓	36% 31%	↑	19% 22%	[4]
Germany (former East)	1990/92 1998	↔	No change	↑	21% 29%	[5]
Germany (National)	1985 2002	↓	no data no data	↑	no data no data	[6]
Italy (National)	1980 1990/91 1994	↓	60% 41% 37%	↑	18% 20% 20%	[7]
Lithuania (MONICA-Kaunas)	1983/85 2001/02	↔	38.3% 41%	↑	4.1% 11%	[8]
Poland (MONICA-Tarnobrzeg)	1983/84 1992/93	↓	58% 54%	↑	11% 21%	[2]
Russia (Novosibirsk)	1985/86 1995	slight ↓	61% 60%	↑	4% 6%	[2]
Spain (MONICACatalonia)	1986/88 1994/96	↓	52% 47%	↑	15% 24%	[9]
Spain (Catalonia)	1982 1998	↓	58% 44%	↑	20% 31%	[10]
Switzerland (National)	1992/93 1997	↑	37% 39%	↑	24% 28%	[11]
Yugoslavia (Novi Sad)	1984 1994/95	↑	48% 49%	↑	26% 30%	[2]
France (MONICA-Toulouse)	1985 1994/97	↓	37% 24%	↔	19% 21%	[12]
Czech Republic (MONICA)	1985 1997/98	↓	49% 37%	↔/slight ↓	28% 26%	[13]
Belgium (National)	1963 1993	↓	~70 ~30	slight ↓	~25 ~20	[14]
Denmark (Copenhagen)	1964/74 1990/94	↓	72% 54%	↓	52% 46%	[15]
Iceland (MONICA)	1983 1993/94	↓	27% 23%	↓	40% 31%	[2]
Ireland (National)	1983 1992	↓	~70 ~30	↓	~40 ~30	[14]
New-Zealand (Auckland)	1982 1993/94	↓	29% 17%	↓	25% 14%	[2]
Sweden (Göteborg)	1985/86 1994/96	↓	32% 26%	↓	34% 29%	[2]
